# Supplementary material for: Deletion of wfs1 Impairs Oligodendrocyte Precursor Cells Dorsal Distribution and Myelination Through the wfs1-hmgcs1 Axis in Zebrafish
Source: Biology (Basel). 2026 Aug 16;15(16):1402. doi: 10.3390/biology15161402 (PMC13510151; doi:10.3390/biology15161402)
Supplement: Supplementary file 1 [file biology-15-01402-s001.zip › File S1-Transcriptome sequencing data and differentially expressed genes (DEGs) in wfs1-- zebrafish at 5 dpf/2.KEGG/wfs1KOvsWT/UP/src/dre04540.html]

dre04540


- dre:100006675

- Up regulated genes

100006675(1.63301233543285)

- dre:323509

- Up regulated genes

323509\_1(2.61236898874666)

- dre:559806

- Up regulated genes

559806(2.30033119315707)

- dre:568726

- Up regulated genes

568726(1.14419230813088)
- dre:560807

- Up regulated genes

560807(1.71247301071773)

- dre:431777

- Up regulated genes

431777(1.35055689210441)

- dre:796490

- Up regulated genes

796490\_2(1.58582846768856)
- dre:103909601

- Up regulated genes

103909601(4.16535952448207)

- dre:323509

- Up regulated genes

323509\_1(2.61236898874666)

Close
